# Monitoring physical functioning as the sixth vital sign: evaluating patient and practice engagement in chronic illness care in a primary care setting--a quasi-experimental design

**DOI:** 10.1186/1471-2296-13-29

**Published:** 2012-04-03

**Authors:** Julie Richardson, Lori Letts, David Chan, Alexis Officer, Sarah Wojkowski, Doug Oliver, Ainsley Moore, Lisa McCarthy, David Price, Sarah Kinzie

**Affiliations:** 1 School of Rehabilitation Science, Faculty of Health Science, Institute of Applied Health Sciences, Room 403, 1400 Main Street West, Hamilton, ON L8S 1C7, Canada; 2 Department of Family Medicine, Faculty of Health Science, McMaster University, McMaster Innovation Park, 175 Longwood Road South, Suite 201A, Hamilton, ON L8P 0A1, Canada; 3 McMaster Family Health Team, Stonechurch Family Health Centre (site), 1475 Upper Ottawa, Hamilton, ON L8W3J6, Canada; 4 McMaster Family Health Team, McMaster (site), 690 Main Street West, Suite A, Hamilton, ON L8S 1A4, Canada

**Keywords:** Rehabilitation, Primary care, Chronic disease, Physical functioning, Self-management, Self-monitoring

## Abstract

**Background:**

In Canada, one in three adults or almost 9 million people report having a chronic condition. Over two thirds of total deaths result from cardiovascular disease, diabetes, cancer and respiratory illness and 77% of persons ≥65 years have at least one chronic condition. Persons with chronic disease are at risk for functional decline; as a result, there is an increased awareness of the significance of functional status as an important health outcome. The purpose of this study was to determine whether patients who receive a multi-component rehabilitation intervention, including online monitoring of function with feedback and self-management workshops, showed less functional decline than case matched controls who did not receive this intervention. In addition, we wanted to determine whether capacity building initiatives within the Family Health Team promote a collaborative approach to Chronic Disease Management.

**Methods:**

A population-based multi-component rehabilitation intervention delivered to persons with chronic illnesses (≥ 44 yrs) (n = 60) was compared to a group of age and sex matched controls (n = 60) with chronic illnesses receiving usual care within a primary healthcare setting. The population-based intervention consisted of four main components: (1) function-based individual assessment and action planning, (2) rehabilitation self-management workshops, (3) on-line self-assessment of function and (4) organizational capacity building. T-tests and chi-square tests were used for continuous and categorical variables respectively in baseline comparison between groups.

**Results:**

Two MANOVA showed significant between group differences in patient reported physical functioning (Λ = 0.88, F = (2.86) = 5.97. p = 0.004) and for the physical performance measures collectively as the dependent variable (Λ = 0.80, F = (6.93) = 3.68. p = 0.0025). There were no within group differences for the capacity measures.

**Conclusion:**

It is feasible to monitor physical functioning as a health outcome for persons with chronic illness in primary care. The timeline for this study was not sufficient to show an increase in the capacity within the team; however there were some differences in patient outcomes. The short timeline was likely not sufficient to build the capacity required to support this approach.

**Trial registration:**

NCT00859638

## Background

Chronic disease is the leading cause of death (60%) and disability (43%) worldwide [1,2]. In Canada, one in three adults or almost 9 million people report having at least one of seven high impact, high prevalence chronic conditions and 77% of persons ≥65 years have at least one chronic condition [3]. Over two thirds of total deaths result from cardiovascular disease, diabetes, cancer and respiratory illness. Canadians with chronic conditions account for over 70% of all nights spent in hospital and half of Canadians with multiple chronic conditions report moderate to severe disability in daily living [3,4]. Optimizing and preserving physical functioning is a central goal for all persons with chronic illness. The results of the Medical Outcomes study of ambulatory patients (mean age 46 years) showed that persons with eight out of the nine chronic conditions studied had worse function across physical, social and mental domains compared to persons without the conditions [5]. Comorbidity was associated with greater decrements in functioning [5].

There is increasing awareness of the importance of functional status as a major heath outcome as well as an emphasis on cost effective interventions for its enhancement [6,7]. There is growing evidence that functional status data are vital to clinical practice and substantiate health system performance [8]. Functional status as an outcome of care is a major concern for persons of all ages with chronic illness who are trying to self-manage their conditions. Loss of self-management abilities has been associated with loss in different functioning domains, including physical functioning [9]. Changes in physical functioning are a better predictor of loss of self-management skills than chronological age. Self-management skills associated with function are needed by persons with chronic disease to prevent and manage functional decline and loss of resources [10]. There is a lack of documentation in health records in hospital and primary care settings about the functional status of patients [7]. It has been suggested that functional status should be the sixth vital sign monitored, in addition to the conventional vital signs of temperature, pulse, respiration rate, blood pressure, and blood oxygen saturation [7]. Physicians do not routinely monitor or assess functional status as part of a patient's management even when prompted [11,12]. Thus, providing awareness about the relationship between physical function and the patient's overall health status in addition to assisting physicians and other members of the health care team to build their capacity in assessing functional status is likely to improve the care for persons with chronic illness. Emerging technology such as electronic health records linked to in situ technology will assist in understanding the dynamic nature of chronic diseases, the management to maximize health and the efficient use of healthcare resources in this population [13].

The Chronic Care Model (CCM) http://www.improvingchroniccare.org is an approach to planned, proactive, population-based, evidenced based and patient-centered care to manage chronic illness rather than reactive acute orientated care [14-16]. The six dimensions of the CCM are organization of health care (health and design systems), clinical information systems, self-management support, delivery system support, and community resources. It represents enhancements in organizations and practices that contribute to productive interactions between providers and patients [16,17]. Effective care using this model requires regular interaction between caregivers and patients and the Institute of Medicine (IOM) recommends alternate methods of interaction rather than solely face-to-face visits [15]. The Expanded Chronic Care Model (ECCM) enlarges the community portion of the CCM and demonstrates how the patient fits within the concepts of population health [17]. This model forms the basis for the framework for chronic disease management in Ontario [17]. Canada has adopted the population health approach as a health care strategy for health policy and program development [18-20].

Allied health professionals provide services to approximately 18% of patients within primary care settings [3]. However physiotherapists and occupational therapists have not been integrated into most primary health care settings in Canada and there is a gap in rehabilitation service provision for persons with chronic illness. This study examined a population-based approach that involves an intervention targeted at patients' self monitoring of physical functioning and capacity building within a primary care setting to support the integration of physical functioning within the Chronic Care Model.

The overall goals for the project were:

1. To assess whether adopting a population-based, rehabilitation self-management approach that focused on physical functioning as a major health outcome in a primary care setting improves the process and outcome of care for patients with chronic conditions.

2. To evaluate the extent to which members of a Family Health Team (FHT) integrate the assessment and monitoring of physical function, and implement of interventions to maintain physical function of their patients within the process of delivering chronic illness care.

Research objectives:

1. To determine whether patients who received a multi-component intervention, namely rehabilitation assessment, action planning, access to online functional assessment with feedback and rehabilitation self-management workshops, showed less functional decline as measured by the Physical Functioning Inventory [21], the Two Minute Walk Test, [22] Grip Strength [23] and lower extremity function tests [24] than case matched controls who did not receive the intervention.

2. To determine whether a multi-component intervention increased patient self-management within a Family Health Team as measured by the Self-Efficacy for Chronic Disease Scale [25] and the Rapid Assessment of Physical Activity [26].

3. To determine whether capacity building initiatives within the FHT increased the collaborative approach to Chronic Disease Management by the Patient Care Provider (PCP) as measured by The Assessment of Primary Care Resources and Supports for Chronic Disease Self-Management (PCRS) [27] and the Patient Assessment of Chronic Illness Care (PACIC) [28].

## Methods

### Study design

**Figure 1:**
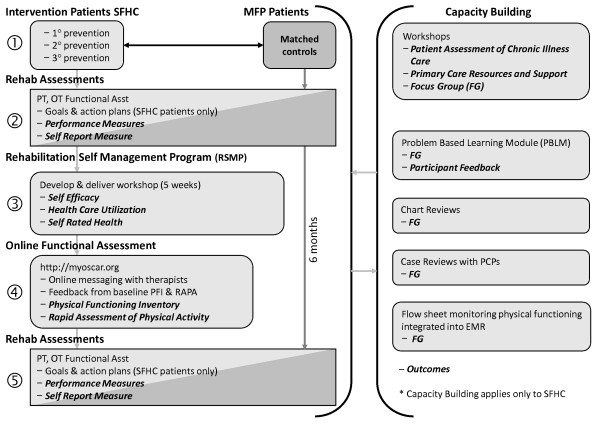
**Population-Based Model for Rehabilitation in Primary Care for Persons with Chronic Illness**.

A before-after design was used to compare a population-based rehabilitation intervention delivered to persons with chronic disease compared to a group of age and sex matched controls receiving usual care. The population-based intervention consisted of four main components: (1) function-based individual assessment and action planning, (2) rehabilitation self-management workshops, (3) on-line self-assessment of function (4) organizational capacity building. These are described below and summarized in Figure 1. Outcome measures for each of the four components of the intervention were administered at baseline and 6 months follow-up. The study was conducted with approval from the Hamilton Health Sciences/McMaster University Research Ethics Board (REB #08-177).

### Sample

Two academic family practice units, the Stonechurch Family Health Centre (SFHC) and the McMaster Family Practice (MFP) that together comprise the McMaster Family Health Team (FHT), participated in the project. Family Health Teams are health care organizations that include a team of family physicians, nurse practitioners, registered nurses, social workers, dietitians, and other professionals who work together to provide health care for their community [29]. The intervention group was recruited from the Stonechurch Family Health Centre (SFHC), with 15 physicians and 30 residents on three teams that serve approximately 13,500 patients. The control group was selected from the McMaster Family Practice (MFP), which provides care to 12,000 patients, attended by 13 physicians and 36 residents. The sites are very similar in composition and organization and operate as one Family Health Team although they are geographically at different sites and have different Medical Directors. Ten family physicians and ten allied health team members from SFHC agreed to participate in the capacity building component of the study. The study used a convenience sample of providers. Patients were randomly selected from the practice database who met the following inclusion criteria: age ≥44 years; having one or more chronic conditions; at least 3 physician visits in the past year; able or willing to access an email address. The ICD-9 billing codes for these chronic diseases were used to select the sample: rheumatoid arthritis, back pain, cardiac arrest, heart failure, chronic obstructive pulmonary disease, stroke, diabetes, emphysema, hypertension, multiple sclerosis, osteoarthritis, osteoporosis, Parkinson's disease, and cerebral vascular accident. Case matched controls, matched on age, gender, type and number of chronic conditions selected from the McMaster Family Practice formed the control group. Control group participants received baseline and final physical functioning assessments from the PT and OT and usual care from their health care team. Sixty patients were recruited to both the intervention and control groups. We recruited 20 patients from three defined stages of functional ability. The levels of functioning were: (1) no difficulty in physical functioning but the patient has made modifications to the tasks they complete or have changed the frequency with which they complete these tasks; (2) early changes or difficulty in physical functioning; (3) established difficulty in physical functioning, experiencing significant or longstanding difficulties with physical functioning, mobility or activities of daily living [30]. The study coordinator, a physiotherapist, reviewed each patient's Electronic Medical Record (EMR), and assigned a physical functioning rating based on the patient's age, number and type(s) of chronic condition(s), length of time chronic conditions were present, work status, disability insurance, mobility status, activities of daily living, use of gait aids, recent surgeries and hospitalizations, use of a disability parking pass, falls, Worker Safety and Insurance Board claims, level of physical activity and smoking. The patient's physician agreed with the investigators' rating in 88% of the cases, the final level was determined by the physician's rating.

### Assessments

#### Evaluation of physical functioning

The Physical Functioning Inventory (PFI) was designed to assess pre clinical functional impairment and physical functioning in older adults [21]. It contains 21 items from 4 different subscales: activities of daily living (ADLs), instrumental activities of daily living (IADL), mobility activities, and moderate/strenuous activities. Probe questions are used to determine whether the person experiences difficulty in completing a task, and changes the method and/or frequency of task performance. Agreement between raters in at least 19 of 22 tasks on the probes has been reported with the PFI to be greater than 80% [21]. The 2-Minute Walk test is a reproducible measure of functional exercise capacity that is measured as the distance walked in 2 min [22]. Correlations between 2- and 6- min walk tests indicate that they are similar measures of exercise tolerance (r = 0.89) [22]. Test-retest reliability ranges from 0.82 to 0.89 in frail elderly individuals. Grip strength was measured using a JAMAR hand-held dynamometer averaged over 3 trials with each hand. It is an indicator of overall strength and a strong predictor of mortality in adults 65-90 years with moderate to severe disabilities [23]. Lower extremity function was measured by the 8-foot timed walk test and standing balance test. The performance of each of these tests and a summed score over several tests, have been associated with self-reported disability [24]. The Timed Up and Go (TUG) was completed only during the patients' final assessment. The TUG is designed to measure balance and basic functional mobility [31]. It is the time in seconds to rise from a regular chair with arms, walk 3 m at a comfortable safe pace, turn, walk back to the chair and sit down. High sensitivity (87%) and specificity (87%) for the TUG's ability to identify community dwelling adults at risk for falls has been reported [32,33]. The inter-rater reliability is 0.98 [31].

#### Evaluation of Rehabilitation Self-Management (RSMP)

Three measures, developed at Stanford Patient Education Research Centre for Chronic Disease, [33] were used to evaluate the workshops. The Self Efficacy for Chronic Disease Scale (SECDS) has 6 items rated on a 10-point scale [24,34,35]. It covers the domains of symptom control, role functioning, emotional functioning and communicating with physicians. The internal consistency ranged from 0.8-0.9 and test re-test reliability r = 0.7-0.9 [24]. The Health Utilization Scale, a 4 item questionnaire that asks about the emergency room use, hospital use and physician visits in the past 6 months, [34-36] and a single question on self-rated health were also administered the first and last day of the program [34]. The Rapid Assessment of Physical Activity (RAPA) was considered an indication of self-management for this study since it was postulated that patients in the intervention group might increase their level of physical activity as a result of increased awareness about physical functioning. The RAPA is a nine-item self-report measure that assesses frequency and intensity of aerobic physical activity as well as frequency of strengthening and flexibility exercises. It has been validated in adults older than 50 years [26]. The RAPA is more highly correlated (r = 0.54) with moderate caloric expenditure than two other commonly used measures of physical activity, the Patient-Centred Assessment and Counselling for Exercise (PACE) (r = 0.44) and the Behavioural Risk Factor Surveillance System (BRFSS) (r = 0.40) [26,37]. It has a reported sensitivity (81%) and positive predictive value (77%) [26].

#### Evaluation of capacity building (CB) intervention

##### Process measures

The Assessment of Primary Care Resources and Supports for Chronic Disease Self-Management (PCRS) was used to evaluate the CB intervention [27]. It assesses patient self-management based on the process and structural level of care. It has acceptable psychometric properties; Cronbach's alpha for individual and organizational support subscales were 0.94 and 0.90 indicating a high level of internal consistency [27]. The Patient Assessment of Chronic Illness Care (PACIC) assesses the patient's perspective on chronic illness care received based on the Chronic Care Model [27,35]. It has 5 scales and an overall summary score. It has demonstrated moderate test-retest reliability (r = 0.58 over 3 months) and correlated moderately with measures of primary care and patient activation (r = 0.32-0.60, median 0,50 p < 0.001) [35].

##### Focus group

A focus group evaluation was held at the end of the project to investigate further the Patient Care Providers' (PCPs') experiences of the CB process, and how this influenced their practice. It was also used to discuss whether the results of the functional assessments were incorporated into their discussions with the patient and the care plan.

### Patient intervention

#### Function-based individual assessment and action planning

The intervention is depicted in Figure 1. At entry to the study, the patient engaged in a joint physiotherapy (PT) and occupational therapy (OT) assessment to identify their concerns and goals related to physical function. The PT and OT conducted a performance-based assessment of physical functioning [22,23,38] using standardized tests and the results were discussed with the patient in the context of age- and sex-based norms. During the initial assessment, patients participated in collaborative goal setting with the therapist, identifying a personal functional goal for the next 3-6 months. Patients then created weekly action plans related to the goal using a self-management behaviour that addressed the functional goal. A copy of the action plan was given to the patient. The therapists documented the results of the performance tests, the problem list and action plan in the EMR for the PCPs to review. The patients received the intervention for a period of 6 months, and then were reassessed by the PT and OT.

#### Rehabilitation self-management workshops

An evidence-based 5-week Rehabilitation Self-Management Program (RSMP) was delivered to increase patients' self-management behaviours and self-efficacy in the monitoring of physical functioning. The goals of the program were to build self-management skills, identify goals, set action plans and engage in problem solving approaches in a group setting. It was a derivative of the Stanford model of Chronic Disease Self-Management Program [39] but was based on rehabilitation principles aimed at maintaining physical function. The topics of the workshops included: changes in physical functioning associated with various stages of aging; the impact of various impairments; managing fatigue and relaxation techniques; the effect of different environments on functioning; how to assess changes in one's function and mobility; how to promote and maintain function including the use of specific exercise and activities; approaches to safety, falls and injury prevention; the use of assistive devices; coping with the impact of pain on mobility and function; the effect of impaired balance on mobility and function; and the effect of medications on mobility and function. All participants who attended the RSMP also received a Personal Health Record (PHR) booklet [40], which they learned to use as part of their self-management to record details of physical functioning and other aspects of their health.

#### On-line self-monitoring of function

The purpose of on-line self-monitoring of function was to increase individual patients' knowledge of their functional status. Patients were able to complete self-assessments of physical functioning, the Physical Functioning Inventory (PFI) [21] and the Rapid Assessment of Physical Activity (RAPA), independently [26]. The assessments were available on MyOSCAR, a web-based system http://myoscar.org. It is a secure personal health record with a secure messaging system for patients to view the results of their online assessments and to communicate with the therapists. The patient was sent a message to their email address indicating when there were updates in the personal health record. The study coordinator summarized the results of the assessment in terms of age- and sex-based norms within the record and responded to the participants' questions using the messaging system. The results of these assessments were also documented in the patient's EMR. Patients were asked to complete the assessments online three times over the course of the study.

### Organizational capacity building

The goal of the capacity building (CB) was to develop PCP skills and organizational supports to address physical functioning as a major health outcome. Monitoring of physical function was prioritized for persons with chronic illness by promoting increased collaboration between 20 PCPs and patients. The capacity building targeted the improvement of chronic illness care through increased understanding of self-management principles. Capacity building was undertaken through a series of activities including: (i) two workshops, (ii) a problem-based learning module, (iii) case reviews of selected patients participating in the study, and (iv) development of a flow sheet for monitoring changes in physical functioning (PF) to be used within the EMR.

#### Workshops

The first workshop introduced SM strategies for chronic disease and the second workshop introduced strategies to monitor PF and provide SM support. The PCPs were educated on how to use the flow sheet to monitor changes in physical functioning and how to integrate this within the patient's assessment.

#### The problem based learning module

"Physical Functioning in Patients with Chronic Disease: the Sixth Vital Sign?" was developed for use in a problem based tutorial. The module was based on a patient case at three different stages of functional decline. The objectives of the module were to examine the impact of chronic disease on physical functioning, rapidly identify patients at high risk of developing a disability, monitor the patient's functional status over time, provide self-management support in the area of physical functioning, explore the role of various team members in this area, and participate in proactive, preventative strategies with an interdisciplinary team.

#### Case reviews

The study coordinator met with PCPs to review the patients' progress in the study activities and to involve them in the process of supporting the patient in the SM of physical functioning. Information about the patients' level of physical functioning, self-identified goals and problem lists, self-management skills and performance test results were discussed with the PCP. The study coordinator discussed what actions the PCP would take as a result of this information and what the coordinator could do to support the patient.

#### Development of a self-management flow sheet

To encourage the monitoring of physical functioning, a decision support and tracking flow sheet that focused on physical functioning was developed for use within the EMR by the authors. Level of physical activity, falls history and Timed Up & Go (TUG) results were included [31]. Information boxes turn red or yellow if the data entered does not meet recommended parameters. To provide decision support, a colour coding system cued the PCP to possible actions if the patient's results were outside the parameters. A list of actions was provided for PCPs to consider including various referral sources. The PCP is notified of areas that require reassessment within 6 months.

### Analysis

T-tests and chi square tests were used for continuous and categorical variables respectively in the baseline comparison between groups. Paired t-tests were used to assess whether there was a significant difference between baseline and follow-up scores for the intervention group on the Self Efficacy for Chronic Disease Scale, Health Care Utilization and Self-Rated Health measures. Paired t-tests were used to assess whether there was a significant difference between baseline and follow-up scores on the PACIC and the PCRS. One-way analysis of variance (α = .05) was used to test for differences between groups in each of the following variables: RAPA, PACIC, Self Efficacy for Chronic Disease Scales, Self-Rated Health. We used a one-way multivariate analysis of covariance (MANOVA) to compare the group effect for the performance measures which included: the two-minute walk test, grip strength, eight foot walk and balance using age as a covariate. We also used a MANOVA to examine group effect for the measure of physical activity, the RAPA and the Physical function Inventory including age as a covariate. STATA/IC 10.1 for Macintosh was used for the analysis [41].

## Results

**Figure 2:**
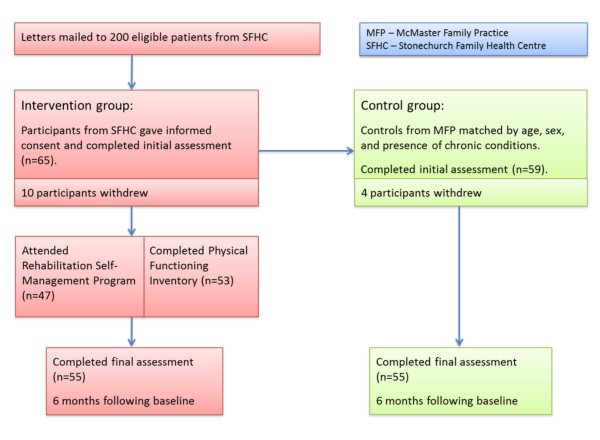
**Flow of patients through the study**.

A total of 200 participants from SFHC were eligible; 65 people (32.5%) agreed to participate in the study and 55 patients completed the final assessment in both the intervention and control group (Figure 2). There were no differences between the two groups at baseline on the variables assessed (Table 1).

**Table 1:** Baseline Characteristics for Intervention and Control Group

	Intervention group (n = 60)	Control group (n = 59)	t/X^2^	P
Age, years mean (SD)	62.78 (11.04)	62.62 (10.29)	t = 0.08	P = 0.93

Gender, mean (SD)				
Female	42 (70%)	43 (72.9%)	X^2 ^= 0.16	P = 0.69
Male	18	17		

Level of function, frequency (%)				

Level 1	22 (36.67%)	22 (33.90%)	X^2 ^= 0.0	P = 0.10
Level 2	20 (33.33%)	20 (36.21%)		
Level 3	18 (30%)	16 (27.12%)		

Use of gait aid, frequency (%)				
No Gait aid	55 (91.67%)	51 (87.93%)		P = 0.71**
Cane	4 (6.67%)	5 (8.62%)		
Walker	1 (1.67%)	2 (3.45%)		

Number of chronic conditions, frequency (%)				

1	21 (35%)	14 (23.72%)	X^2 ^= 6.22	P = 0.18
2	26(43%)	22 (37.29%)		
3	9 (15%)	18 (30.51%)		
4	3 (5%)	5 (8.47%)		
5	1 (1.66%)	0		

Self-Rated Health, mean (SD) (1:Excellent - 5:Poor)	2.94 (0.74)	3.05 (1.13)	t = -0.60	P = 0.55

Self-Efficacy for Chronic Disease Scale, mean (SD) (max score = 60)	6.92(1.84)	6.83 (2.24)	t = 0.22	P = 0.83

Physical Activity Level (1:Sedentary-7:Active), mean (SD)	4.58 (1.61)	4.32 (1.79)	t = 0.84	P = 0.40

*Chi-square tests and *t*-test were used for categorical and continuous variables respectively

**Fishers exact test

At follow-up, there were no significant difference between groups on the PFI overall score or subscale scores (Table 2). The ANOVA showed a significant difference between the groups in the level of physical activity as measured by the RAPA. The MANOVA showed a significant difference between the groups for physical function (PFI) and physical activity (RAPA) (Λ = 0.88, F = (2.86) = 5.97. p = 0.004). There was no significant difference between groups on Self-Rated Health or Self-Efficacy for Chronic Disease Scale scores (Table 2).

**Table 2:** Results of Self-Report Measures by Group: Physical Functioning Inventory, Rapid Assessment of Physical Activity, Self-Rated Health and Self-Efficacy for Chronic Disease

	Intervention		Control		F	P
	**Baseline** **Mean (SD)**	**Follow-up**	**Baseline**	**Follow-up**		

Physical Functioning Inventory Overall Score	81.85 (19.74)	85.89 (17.13)	79.39 (20.43)	83.07 (18.36)	0.64	0.42

ADL Subscale	21.76 (4.88)	22.68 (4.07)	20.97 (5.01)	21.96 (4.30)	0.77	0.38

IADL Subscale	22.53 (3.60)	22.74 (3.43)	21.90 (4.44)	23.19 (2.57)	0.42	0.52

Mobility Subscale	20.28 (5.44)	21.08 (5.21)	19.57 (5.5)	19.87 (5.50)	1.28	0.26

Moderate Activity Subscale	17.45 (9.46)	20.2 (7.52)	15.61 (10.43)	17.5 (10.06)	2.33	0.13

Level of Physical Activity - RAPA (1: Sedentary-7: Active)	4.58 (1.61)	5.09 (1.47)	4.32 (1.79)	4.05 (1.58)	12.95	0.0005

Self-Rated Health (1:Excellent - 5: Poor)	2.94 (0.74)	2.87 (0.85)	3.05 (1.13)	3.03 (0.94)	0.83	0.36

Self-Efficacy for Chronic Disease	6.92 (1.84)	7.44 (1.66)	6.83 (2.24)	7.04 (2.33)	1.6	0.31

The Self-Efficacy for Chronic Disease Scale score ranges from 0-10, with lower scores indicating lower confidence at completing tasks or managing symptoms related the chronic illness. Self-Rated Health is rated on a scale from 1-excellent, to 5-poor

Forty six participants completed the online self-monitoring of physical function independently at baseline and 33 at follow-up using MyOSCAR. Seven more patients completed the follow-up with support of the study coordinator. The average amount of time spent assisting patients who did not use online methods independently was approximately 1.5 h per patient over the course of the study. Although 48 out of 60 (80%) of the intervention group participants had access to an email address and were accustomed to using online services, several of these participants required support.

There was a significant difference between the groups in favour of the intervention group in grip strength (both dominant and non-dominant hands) (Table 3). The MANOVA also showed a significant between group effect when the physical performance measures were examined collectively as the dependent variable (Λ = 0.80, F = (6,93) = 3.68. p = 0.0025). In the one way ANOVA grip strength was the only outcome that showed a between group difference.

**Table 3:** Results of Performance Measures by Group: Grip Strength, Walk Tests and Balance

Outcome Measure	Intervention Group		Control Group		F	P
	**Baseline** **Mean (SD)**	**Follow-up**	**Baseline**	**Follow-up**		

Dominant hand grip strength	26.9 (12.4)	30.59 (11.29)	22.92 (11.82)	23.01 (11.49)	11.96	0.0008

Non-dominant hand grip strength	24.75 (12.7)	28.60 (11.98)	21.67 (11.05)	20.63 (10.40)	13.41	0.0004

Two-minute walk test	152.09 (40.62)	157.27 (39.12)	151.93 (36.29)	167.34 (28.79)	2.13	0.53

Eight foot walk test	2.29 (0.92)	2.35 (0.55)	2.32 (0.9)	2.43 (0.72)	0.39	0.53

Balance test	3.56(0.85)	3.759 (0.61)	3.5 (0.84)	3.83 (0.55)	0.40	0.53

TUG	--	9.66 (3.21)	--	9.69 (2.71)	0.01	0.94

There was no significant difference between the groups for Health Care Utilization (visits to the physician, emergency room visits and hospitalizations) (Table 4).

**Table 4:** Results of Health Care Utilization by Group: Physician Visits, Emergency Room Visits, Hospitalisations

	Intervention		Control		F	P
	**Baseline** **Mean (SD)**	**Follow-up**	**Baseline**	**Follow-up**		

Number of physician visits	4.88 (7.29)	4.24 (6.073)	3.55 (3.12)	4 (4.29)	0.05	0.82

Number of emergency room visits	0.19 (0.99)	0.22 (0.57)	0.23 (0.50)	0.24 (0.82)	0.02	0.89

Number of hospitalizations	0.19 (0.86)	0.05 (0.23)	0.19 (0.97)	0.07 (0.33)	0.11	0.73

Number of total nights spent in hospital	0.28 (0.17)	0.2 (1.11)	0.15 (0.11)	0.25 (1.24)	0.06	0.81

There were no significant differences or within group change in the capacity building outcomes or the Primary Care Resources and Supports scores (Table 5). The Patient Assessment of Chronic Illness (PACIC) also showed no significant differences for between group change as a result of CB initiatives (Table 6). The PACIC was used to measure changes in practice from the patient's perspective as a result of capacity building initiatives.

**Table 5:** Results Primary Care Resources and Supports (PCRS)

	Baseline (n = 8)	Follow-up (n = 8)	t	P
Patient support subscale	45 (12.15)	47.06 (8.33)	-0.86	0.42

Organizational support subscale	41.25 (11.39)	46.81 (6.15)	-1.78	0.12

**Table 6:** Results of Patient Assessment of Chronic Illness Care (PACIC) with Subscale Scores by Group

Outcome Measure	Intervention		Control		F	p
	**Baseline**	**Follow-up**	**Baseline**	**Follow-up**		

PACIC overall score	2.57 (0.17)	2.9 (1.11)	2.52 (0.11)	2.7 (0.91)	0.91	0.34

Patient activation Subscale	2.87 (1.42)	3.36 (1.33)	3.11 (1.34)	3.29 (1.22)	0.07	0.79

Delivery system design/decision support subscale	3.24 (0.15)	3.24 (1.10)	2.98 (0.14)	2.98 (1.03)	1.57	0.21

Goal setting subscale	2.36 (0.19)	2.67 (1.31)	2.31 (0.13)	2.56(1.06)	0.22	0.64

Problem solving/contextual counseling subscale	3.02 (0.20)	3.14 (1.24)	3.07 (0.15)	3.13(1.22)	0.00	0.98

Follow-up/coordination subscale	2.44 (0.16)	2.44 (1.19)	2.13 (0.31)	2.13 (0.96)	2.06	0.15

Self-Rated Health and Self-Efficacy for Chronic Disease were administered to the intervention group before and after the Rehabilitation Self-Management Program. There was a significant within group difference for the intervention group in Self-Efficacy for Chronic Disease Scale scores. These scores increased by 0.54 points after RSMP, p = 0.01 (Table 7). In the RSMP, the mean number of sessions attended was 3.45; 75% of participants attended ≥3/5 sessions.

**Table 7:** Mean Scores on Outcome Measures to evaluate RSMP (Intervention Group): Self-Rated Health and Self-Efficacy

Outcome measure	Pre RSMP Mean (SD)	Post RSMP	Mean Difference	t	P
Self-Rated Health	2.92 (0.75)	2.97 (0.73)	0.05	-0.60	0.55

Self-Efficacy for Chronic Disease Scale	7.03 (1.76)	7.57 (1.59)	0.53	-2.54	0.01

### Results of focus group

Nine PCPs from a variety of disciplines attended the focus group. Several PCPs stated they became more intentional in addressing the patients' physical functioning as a result of the CB workshops. They reported an increase in the level of the patients' physical activity, improved goal setting and problem solving, and greater focus in their interactions with PCPs. PCPs felt their own efforts at integrating the self-management aspects of care could be improved. The main barrier identified by the PCPs was the lack of time to address the multiple concerns that patients with chronic conditions face and SM goals were sometimes given a lower priority.

### Results of capacity building process measures

Forty-eight PCPs from SFHC participated in the capacity building initiatives. Thirty three PCPs from 6 different disciplines attended the workshops (Table 8) and 16 individuals, which included a group of residents and a group of allied health professionals attended the PBLM. The residents noted that specific tools and handouts within the module would assist them in applying the information in clinical practice.

**Table 8:** Disciplines Represented in Capacity Building Initiatives

Discipline	Workshop 1	Workshop 2	PBLM*	Case Reviews	Focus Group
Physician	5	6	1	5	3

Medical Resident	8	7	10	-	

Nurse Practitioner	2	3	4	1	3

Registered Dietitian	1	-	-	1	

Social Worker	1	1	-	1	2

Reg. Practical Nurse	1	-	-	-	1

Pharmacist	-	-	1	-	

Total	18	15	16	8	9

*PBLM: Problem based learning module

### Results of case review process

Case reviews about patients' physical functioning during the study were undertaken with 8 PCPs. The PCPs discussed whether they would take a specific action as a result of the case review (e.g. referral to a community program or a CCAC day program, a Chronic Pain Program or a dietitian) as well as plans for ongoing support for self-management activities with the participant. The PT met with PCPs to review a total of 21 patient cases. Each case took approximately 10 min to complete.

## Discussion

The purpose of this study was to determine whether a population-based rehabilitation intervention for persons with chronic conditions in a primary care setting resulted in decreased functional decline and improved self-management compared to case-matched controls. The goal of the capacity building initiatives was to increase PCP collaboration in chronic disease management. Self-efficacy for managing chronic conditions improved as seen by a significant increase in self-efficacy scores immediately after the RSMP. There was greater improvement in the intervention group than in the control group in level of physical activity as measured by the RAPA (p = 0.0005) and bilateral grip strength (p = 0.0008). The current intervention is a feasible method to increase self-efficacy for physical activity through RSMP workshops together with reinforcement from members of the primary care team. This approach has the potential to impact on physical functioning. Counseling by primary care physicians over 6 weeks has been reported to increase levels of physical activity; however this effect was not sustained at 8 months [42]. Sustained improvement in physical activity levels requires more intensive patient contact [43]. Physically active individuals with and without chronic conditions have been reported to be less likely to develop a disability when compared to less active individuals [44]. This population-based approach with a restricted time frame resulted in elevated levels of physical activity.

The improvements in grip strength may be the result of increased levels of physical activity. Grip strength is a measure that reflects generalized muscle strength and acts as a predictor of disability [38]. Older men and women with chronic illness have shown 30% improvement in upper and lower extremity strength as a result of stretching, strengthening and aerobic exercise [45].

The capacity building initiatives were well received with participation from 48 PCPs from 5 disciplines. There was no significant change in the amount or type of information relating to physical functioning that was documented in patients' EMRs by PCPs during the study period. The impact of PCP self-management support on patient care as measured by the PCRS and PACIC did not change in the 6 month time period but focus group results indicated that PCPs recognized the importance of these concepts. Time availability was identified as a significant barrier to collaborating around the maintenance of physical functioning; however PCPs supported the integration of rehabilitation professionals into the FHT to address physical functioning.

The feasibility of the intervention was demonstrated throughout the study. In the RSMP, the mean number of sessions attended was 3.45, while 75% of participants attended ≥3/5 sessions. The attrition rate of 25% in this study was comparable to 28%-50% attrition rates reported in other studies of CDSMP [14,46,47]. These data suggest that retention of participants in this type of program is an ongoing challenge. We tracked the feasibility of using MyOSCAR to administer outcome measures for the self-monitoring of physical function. The average amount of time required over the 6 months to support patients with online questionnaires was 1.5 h/patient. Additional support maybe required for persons who do not access the internet or who do not operate a computer regularly. Healthcare reform includes the use of technology by both health care professionals and patients [13], however it is likely there will always be a gap between the ability of the technology and the ability of some patients to engage in the monitoring or usage. Further understanding of the patients' difficulties in interacting with the technology to maximize this self monitoring approach is required. Recent research has shown that increasing adherence to monitoring health indices such as blood pressure and blood glucose does not necessarily improve health outcomes [48]. It is possible that the technology associated with the intervention might have deterred participants from completing assessments, however similar cohorts in the future will be more experienced with incorporating computer assisted technology into health related management. Future work needs to evaluate whether an increase in adherence to monitoring physical function is accompanied by a concomitant increase in performance, health status or quality of life outcome.

The capacity building strategies to improve self-management support of physical functioning were feasible in the FHT. The PCPs indicated that the workshops increased their awareness of functional status as an important health outcome. Incentives such as Continuing Medical Education (CME) credits for the physicians linked to workshop attendance may have increased participation and a higher rate of participation would have been desirable. Involving PCPs in any educational activity during clinical hours is always going to be a challenge. Multiple strategies and opportunities for participation, as implemented in this study and recommended in knowledge translation approaches, will likely maximize involvement [49]. PCPs reported a lack of time for management and follow-up with patients with complex chronic issues as a barrier to implementing principles discussed in CB initiatives. They did report patients' behavioral changes such as increased levels of physical activity observed in outcome measures such as the RAPA. Improved self-efficacy and increased initiative taken by patients was also noted in those self-managing their conditions. There was no change in health care utilization.

A population-based approach to rehabilitation for persons with chronic illness who had varying levels of physical functioning was successfully implemented in a compressed time period. The current intervention was designed to take a comprehensive approach to improve chronic disease management by addressing the characteristics of the patients as well as the health care environment in which patients receive services. The use of multiple strategies to address barriers to change in PCP behaviour is a strength of this study and over an extended period is likely to effect organizational change [50].

This approach uses limited resources to benefit a larger number of patients than would normally be possible with care by a single provider. A group-based intervention is a feasible way to address the demand for rehabilitation professionals and to provide patients with chronic conditions access to services that may not be available to them [51]. This study generated modest results which would be consistent with other rehabilitation interventions that typically produce small to moderate effect sizes [52]. However this trend is offset by small associated risks, an important consideration in a population with multiple morbidity where many of the interventions have their own associated risks and patients and physicians are required to make judgments about which risks they would prefer to undertake [53,54]. To date, PTs have not been integrated into FHT practices in Ontario [55] while funding for OTs is being implemented by the Ministry of Health and Long Term Care on a case by case basis [55]. With a shift in chronic disease management from disease recovery to functional health, the expertise of rehabilitation professionals in considering the functional consequences of disease can make a valuable contribution to primary care [56].

The principal limitations of this study were the short duration of the study period and the non-randomized design. Six months was likely insufficient time to result in a change in self-rated health, health care utilization and physical functioning. Changes in organizational structure and practices can also take much longer than 6 months to occur [57]. Quality of life maybe a more appropriate outcome of interest than health status since it may take years to change health status through lifestyle interventions. However, it is possible that patients could experience an important improvement in their quality of life as a result of the intervention. Physical functioning is a core component of a person's health status and quality of life. Persons with chronic disease will experience episodic illness which along with the symptoms of a chronic disease is likely to negatively influence their physical functioning. It is imperative that physical functioning is optimized and that the physiological reserve required for daily activities is maintained.

## Conclusion

It is feasible to monitor physical functioning as a health outcome for persons with chronic illness in primary care. The timeline for this study was not sufficient to show an increase in the capacity within the team; however there were some differences in patient outcomes. The short timeline was likely not sufficient to build the capacity required to support this approach. This intervention needs to be tested by a large scale randomized controlled trial.

## Abbreviations

CCM: Chronic Care Model; ECCM: Expanded Chronic Care Model; FHT: Family Health Team; PCP: Patient Care Provider; PCRS: The Assessment of Primary Care Patient Care Resources and Supports; PACIC: Patient Assessment of Chronic Illness Care; SFHC: Stonechurch Family Health Centre; MFP: McMaster Family Practice; PFI: The Physical Functioning Inventory; ADL: Activities of Daily Living; IADL: Instrumental Activities of Daily Living; RAPA: Rapid Assessment of Physical Activity; TUG: Timed Up and Go; SECDS: The Self Efficacy for Chronic Disease Scale; EMR: Electronic Medical Record; PT: Physiotherapy; OT: Occupational Therapy; RSMP: Rehabilitation Self-Management Program; PCP: Patient Care Provider.

## Competing interests

The authors declare that they have no competing interests.

## Authors' contributions

JR, LL, DC and LMC contributed to the design of the study. JR and LL were responsible for obtaining funding for the study. AO and SW participated in the acquisition of data. JR, LL, AO contributed to the analysis and interpretation of the data. DO, AM, LMC, DP, SK provided administrative, technical or material support. JR drafted the manuscript and all authors critically reviewed the manuscript for important intellectual content. All authors approved the final version of the manuscript submitted for publication.

## Pre-publication history

The pre-publication history for this paper can be accessed here:

http://www.biomedcentral.com/1471-2296/13/29/prepub

## References

[B1] Gill T Geriatric medicine: it's more than caring for old people Am J Med 2002 113 85 90 10.1016/S0002-9343(02)01187-7 12106630

[B2] Fitzpatrick E Johnston JC Angus D Durieux-Smith A Fitting audiology within the population health perspective Can J Publ Health 2006 97 153 155 10.1007/BF03405338 PMC6976015 16620007

[B3] Health Council of Canada Why health care renewal matters: Learning from canadians with chronic health conditions 2007

[B4] Griffith L Raina P Wu H Zhu B Stathokostas L Population attributable risk for functional disability associated with chronic conditions in canadian older adults Age Ageing 2010 39 738 745 10.1093/ageing/afq105 20810673

[B5] Stewart A Greenfield S Hays RD Functional status and well-being of patients with chronic conditions. Results from the medical outcomes study JAMA 1989 262 907 913 10.1001/jama.1989.03430070055030 2754790

[B6] Institute of Medicine Crossing the quality chasm: A new health system for the 21st century 2001 http://www.nap.edu/html/quality_chasm/reportbrief.pdf Accessed March 25 2012 25057539

[B7] Bierman AS Functional status - the sixth vital sign J Gen Intern Med 2001 16 785 786 11722694 10.1111/j.1525-1497.2001.10918.x PMC1495293

[B8] Romanow RJ Building on values: The future of health care in canada. Final report of the commission on the future of health care in Canada 2004 Ottawa, on: Canadian Government Publishing

[B9] Schuurmans H Steverink N Lindenberg S Frieswijk N Slaets JP Old or frail: What tells us more? Journal of Gerontology Medical Sci 2004 59A 962 965 10.1093/gerona/59.9.m962 15472162

[B10] Steverink N Slaets JP Schuurmans H Van Lis M Measuring frailty: developing and testing the GFI (Gronigen Frailty Indicator) Gerontologist 2001 41 Special Issue 1 236

[B11] Calkins DR Rubenstein LV Cleary PD Failure of physicians to recognize functional disability in ambulatory patients Ann Intern Med 1991 114 451 454 1825267 10.7326/0003-4819-114-6-451

[B12] Calkins DR Rubenstein LV Cleary PD Functional disability screening of ambulatory patients J Gen Intern Med 1994 9 10 590 592 10.1007/BF02599291 7823232

[B13] Kang H Mahoney DF Hoenig H In situ monitoring of health in older adults: Technologies and issues Am Geriatr Soc 2010 58 1579 1586 10.1111/j.1532-5415.2010.02959.x 20646105

[B14] Wagner EH Austin BT Von Korff M Organizing care for patients with chronic illness Milbank Q 1996 74 511 544 10.2307/3350391 8941260

[B15] Wagner EH Chronic disease management: What will it take to improve care for chronic illness? Effective Clin Pract 1998 1 2 4 10345255

[B16] Wagner EH Austin BT Davis C Hindmarsh M Schaefer J Bonomi A Improving chronic illness care: Translating evidence into action Health Aff 2001 20 64 78 10.1377/hlthaff.20.6.64 11816692

[B17] Improving Chronic Illness http://www.improvingchroniccare.org/change/model/components.html Acessed 31/05/2011

[B18] Barr V Robinson S Marin-Link B Underhill L Dotts A Ravensdale D Salivaras S The expanded chronic care model: An integration of concepts and stratgies from population health promotion and the chronic care model Hosp Q 2003 7 73 82 14674182 10.12927/hcq.2003.16763

[B19] Multijurisdictional Collaboration Guiding Facilitation in the Canadian Context: Enhancing Primary Health Care 2006 St John's, NL: Department of Health and Community Services 6

[B20] Health Canada Population Health Promotion: An Integrated Model of Population Health and Health Promotion http://www.phac-aspc.gc.ca/ph-sp/php-psp/php3-eng.php Accessed 26^th ^March 2012

[B21] Whetstone L Fozard J Metter E The physical functioning inventory: A procedure for assessing physical function in adults J Aging Health 2001 13 467 493 10.1177/089826430101300402 11813737

[B22] Butland RJ Pang C Gross E Two-, six-, twelve-minute walking tests in respiratory disease BMJ 1982 284 1607 1608 10.1136/bmj.284.6329.1607 6805625 PMC1498516

[B23] Rantanen T Volpato S Ferruci L Heikkinen E Fried L Guralnik J Handgrip strength and cause-specific and total mortality in older disabled women: Exploring the mechanism J Am Geriatr Soc 2003 51 636 641 10.1034/j.1600-0579.2003.00207.x 12752838

[B24] Guralnik J Simonsick E Ferrucci L Berkman L Blazer G Wallace P A short physical performance battery assessing lower extremity function: Association with self-reported disability and prediction of mortality and nursing admission J Gerontol 1994 2 M85 M94 8126356 10.1093/geronj/49.2.m85

[B25] Lorig K Ritter P Stewart A Sobel D Brown B Bandura A Gonzalez V Laurent D Holman H Chronic disease self-management program- 2 year health status and health care utilization outcomes Med Care 2001 39 1217 1223 10.1097/00005650-200111000-00008 11606875

[B26] Topolski T LoGerfo J Patrick D Williams B Walwick J Patrick M The rapid assessment of physical activity (RAPA) among older adults Prev Chronic Dis 2006 3 4 A118 16978493 PMC1779282

[B27] Brownson C Miller D Crespo R Neuner S A quality improvement tool to assess self-management support in primary care The Joint Commission J Qual Patient Saf 2007 13 408 416 10.1016/s1553-7250(07)33047-x 17711143

[B28] Glasgow R Wagner EH Schaefer J Mahoney LD Reid RJ Greene SM Development and validation of the patient assessment of chronic illness care (PACIC) Med Care 2005 43 436 444 10.1097/01.mlr.0000160375.47920.8c 15838407

[B29] Family Health Teams http://www.health.gov.on.ca/transformation/fht/fht_mn.html Acessed 31/05/2011

[B30] Fried LP Bandeen-Roche K Williamson JD Functional decline in older adults: expanding methods of ascertainment J Gerontol Med Sci 1996 51A M206 M214 10.1093/gerona/51A.5.M206 8808990

[B31] Rossier P Wade D Validity and reliability comparison of 4 mobility measures in patients presenting with neurological impairment Arch Phys Med Rehabil 2001 82 1 9 13 10.1053/apmr.2001.9396 11239279

[B32] Podsiadlo D Richardson S The timed "up and go": a test of basic functional mobility for frail elderly persons J Am Geriatr Soc 1991 39 142 148 1991946 10.1111/j.1532-5415.1991.tb01616.x

[B33] Shumway Cook A Brauer S Wollacott M Predicting the probability for falls in community-dwelling older adults using the timed up & go test Phys Ther 2000 80 896 903 10960937

[B34] Research Instruments Developed, Adapted or Used by the Stanford Patient Education Research Center http://patienteducation.stanford.edu/research Accessed 31/05/2011

[B35] Lorig K Stewart A Ritter P Outcome measures for health education and other health care interventions 1996 Thousand Oaks, CA: Sage Publications

[B36] Ritter P Kaymaz H Stewart A Sobel D Lorig K Self-reports of health care utilization compared to provider records J Clin Epidemiology 2001 54 2 136 141 10.1016/S0895-4356(00)00261-4 11166528

[B37] Patrick K Sallis J Long B Calfas K Wooten W Heath G A new tool for encouraging activity Phys Sports Med 1994 22 45 55 10.1080/00913847.1994.11947706 29275663

[B38] Rantanen T Gurlanik J Foley D Masaki K Leveille S Curb J Handgrip strength and cause-specific and total mortality in older disabled women: Exploring the mechanism JAMA 1999 281 558 560 10.1001/jama.281.6.558 10022113

[B39] Lorig K Sobel D Stewart A Brown B Bandura A Ritter P Gonzalez V Laurent D Holman H Evidence suggesting that a chronic disease self-management program can improve health status while reducing hospitalization: a randomized trial Med Care 1999 37 5 14 10.1097/00005650-199901000-00003 10413387

[B40] McGowan P Permission to Use the Personal Health Record. Personal Communication 2007 Victoria, BC: University of Victoria

[B41] StataCorp. Stata for Macintosh 2008 10.1 Texas: Statacorp.

[B42] Goldstein M Bernardine M Lynn H Jette AM Rakowski W McDermott S Physician-based physical activity counseling for middle-aged and older adults: a randomized trial Ann Behav Med 1991 21 40 47 18425653 10.1007/BF02895032

[B43] Rejeski W Brawley L Ambriosius B Fox L Older adults with chronic disease: benefits of group-mediated counseling in the promotion of physically active lifestyles Health Psychol 2003 22 414 423 12940398 10.1037/0278-6133.22.4.414

[B44] Landi F Onder G Carpenter I Cesari M Soldato MRB Physical activity prevented functional decline among frail community-living elderly subjects in an international observational study Phys J Clin Epidemiology 2007 60 518 524 10.1016/j.jclinepi.2006.09.010 17419963

[B45] Smith T Kennedy S Smith M Orent S Fleshner M Physiological improvement and health benefits during an exercise-based comprehensive rehabilitation program in medically complex patients Exerc Immunol Rev 2006 12 86 96 17201074

[B46] Lorig K Ritter P Laurent D Long-term randomized controlled trial of tailored-print and small-group arthritis self-management interventions Med Care 2004 42 346 354 10.1097/01.mlr.0000118709.74348.65 15076811

[B47] Norris S High K Gill T Hennessy S Health Care for Older Americans with Multiple Chronic Conditions: a research agenda J Am Geriatr Soc 2007 56 1 149 159 18047493 10.1111/j.1532-5415.2007.01530.x

[B48] Grol R Grimshaw J From best evidence to best practice: effective implementation of change in patients' care Lancet 2003 362 9391 1225 1230 10.1016/S0140-6736(03)14546-1 14568747

[B49] Holbrook A Thabane L Keshavjee K Dolovich L Berstein B Troyan S Foster G Gerstein H Investigators for the COMPETE II Investigators: Individualized electronic decision support and reminders to improve diabetes care in the community: COMPETE II randomised trial CMAJ 2009 181 1-2 37 44 10.1503/cmaj.081272 19581618 PMC2704409

[B50] Grimshaw J Shirran L Thomas R Mowatt G Fraser C Bero L Grilli R Harvey E Oxman A O'Brien M Changing provider behaviour: an overview of systematic reviews of interventions Med Care 2001 39 8, suppl 2 2 45 11583120

[B51] Richardson J Letts L Chan D Multidisciplinary approach to primary care not "two tiered" Ontario Med Rev 2007 73 7 9 10

[B52] Wade DT Smeets RM Verbunt J Research in rehabilitation medicine: methodological challenges J Clin Epidemiology 2010 63 7 699 704 10.1016/j.jclinepi.2009.07.010 19788953

[B53] Tinetti ME Health Outcome Priorities Among Competing Cardiovascular, Fall Injury, and Medication-Related Symptom Outcomes J Am Geriatr Soc 2008 56 1409 1416 10.1111/j.1532-5415.2008.01815.x 18662210 PMC3494099

[B54] Fried TR McGraw S Agonstini JV Tinetti ME Views of Older Persons with Multiple Morbidities on Competing Outcomes and Clinical Decision Making J Am Geriatr Soc 2008 56 1839 1844 10.1111/j.1532-5415.2008.01923.x 18771453 PMC2596278

[B55] Ministry of Health and Long Term Care Family Health Teams and Nurse Practitioner-Led Clinics 2009 Toronto: Ministry of Health and Long Term Care

[B56] Rijken P Dekker J Clinical experience of rehabilitation therapists with chronic diseases: a quantitative approach Clin Rehabil 1998 12 143 150 10.1191/026921598669374346 9619656

[B57] Bazzoli GJ Dynan L Lawton RB Yap C Two decades of organizational change in health care: what have we learned? Med Care Res Rev 2004 61 247 331 10.1177/1077558704266818 15358969

